# Supplementary material for: COVID-19 induced birth sex ratio changes in England and Wales
Source: PeerJ. 2023 Feb 17;11:e14618. doi: 10.7717/peerj.14618 (PMC9940645; doi:10.7717/peerj.14618)
Supplement: Supplemental Information 1 [file peerj-11-14618-s001.docx]

**Table S1.** Parameter selection procedure for the autoregressive moving average (ARMA) model to fit and predict secondary sex ratio in England and Wales.

| AR | MA | AR | | | | |  | MA | | | | | AIC |
| --- | --- | --- | --- | --- | --- | --- | --- | --- | --- | --- | --- | --- | --- |
|  |  | L1 | L2 | L3 | L4 | L5 |  | L1 | L2 | L3 | L4 | L5 |  |
| 1 | 0 | -0.01 |  |  |  |  |  |  |  |  |  |  | -897.15 |
| 1 | 1 | 0.92 |  |  |  |  |  | -1.00 |  |  |  |  | -897.39 |
| 1 | 2 | 0.91 |  |  |  |  |  | -0.96 | -0.04 |  |  |  | -897.52 |
| 1 | 3 | 0.89 |  |  |  |  |  | -0.95 | 0.01 | -0.07 |  |  | -893.89 |
| 1 | 4 | 0.71 |  |  |  |  |  | -0.75 | 0.02 | -0.03 | -0.07 |  | -891.4 |
| 1 | 5 | 0.62 |  |  |  |  |  | -0.67 | 0.01 | 0.04 | 0.05 | -0.21 | -891.78 |
| 2 | 0 | -0.01 | 0.02 |  |  |  |  |  |  |  |  |  | -895.18 |
| 2 | 1 | 0.95 | -0.04 |  |  |  |  | -1.00 |  |  |  |  | -897.54 |
| 2 | 2 | -0.01 | 0.86 |  |  |  |  | -0.10 | -0.90 |  |  |  | -896.00 |
| 2 | 3 | NC |  |  |  |  |  | NC |  |  |  |  |  |
| 2 | 4 | NC |  |  |  |  |  | NC |  |  |  |  |  |
| 2 | 5 | 1.30 | -0.77 |  |  |  |  | -1.44 | 0.95 | -0.01 | -0.01 | -0.17 | -899.75 |
| 3 | 0 | -0.01 | 0.02 | -0.02 |  |  |  |  |  |  |  |  | -893.2 |
| 3 | 1 | 0.95 | 0.02 | -0.07 |  |  |  | -1.00 |  |  |  |  | -896.00 |
| 3 | 2 | -1.53 | -0.99 | -0.05 |  |  |  | 1.57 | 1.00 |  |  |  | -893.94 |
| 3 | 3 | NC |  |  |  |  |  | NC |  |  |  |  |  |
| 3 | 4 | -0.08 | -0.02 | -0.74 |  |  |  | 0.02 | 0.09 | 0.88 | 0.16 |  | -894.88 |
| 3 | 5 | 2.23 | -2.03 | 0.75 |  |  |  | -2.40 | 2.41 | -0.94 | -0.18 | 0.12 | -898.86 |
| 4 | 0 | -0.01 | 0.02 | -0.02 | -0.01 |  |  |  |  |  |  |  | -891.21 |
| 4 | 1 | 0.72 | 0.02 | -0.03 | -0.11 |  |  | -0.75 |  |  |  |  | -892.38 |
| 4 | 2 | -0.82 | -0.92 | 0.06 | 0.08 |  |  | 0.83 | 0.97 |  |  |  | -891.28 |
| 4 | 3 | 1.90 | -1.71 | 0.68 | -0.13 |  |  | -2.65 | 2.75 | -1.35 |  |  | -899.42 |
| 4 | 4 | 1.71 | -1.83 | 1.51 | -0.88 |  |  | -1.80 | 2.05 | -1.80 | 1.00 |  | -904.70 |
| 4 | 5 | 1.39 | 0.05 | -1.20 | 0.65 |  |  | -1.23 | 0.03 | 1.05 | -0.68 | -0.16 | -897.78 |
| 5 | 0 | -0.01 | 0.01 | -0.01 | -0.02 | -0.18 |  |  |  |  |  |  | -892.38 |
| 5 | 1 | 0.56 | 0.02 | -0.02 | -0.01 | -0.2 |  | -0.61 |  |  |  |  | -893.52 |
| 5 | 2 | NC |  |  |  |  |  | NC |  |  |  |  |  |
| 5 | 3 | NC |  |  |  |  |  | NC |  |  |  |  |  |
| 5 | 4 | 0.73 | -0.37 | 0.79 | -0.82 | -0.18 |  | -0.90 | 0.47 | -0.84 | 0.96 |  | -903.07 |
| 5 | 5 | NC |  |  |  |  |  | NC |  |  |  |  |  |

AIC: Akaike Information Criterion; AR: autoregressive parameter; MA: moving average parameters; NC: not converged.
